# Asymptomatic Carriers of Toxigenic *C. difficile* in Long-Term Care Facilities: A Meta-Analysis of Prevalence and Risk Factors

**DOI:** 10.1371/journal.pone.0117195

**Published:** 2015-02-23

**Authors:** Panayiotis D. Ziakas, Ioannis M. Zacharioudakis, Fainareti N. Zervou, Christos Grigoras, Elina Eleftheria Pliakos, Eleftherios Mylonakis

**Affiliations:** Infectious Diseases Division, Warren Alpert Medical School of Brown University, Providence, Rhode Island, United States of America; Cleveland Clinic, UNITED STATES

## Abstract

**Background:**

The impact of *Clostridium difficile* colonization in *C. difficile* infection (CDI) is inadequately explored. As a result, asymptomatic carriage is not considered in the development of infection control policies and the burden of carrier state in long-term care facilities (LTCFs) is unknown.

**Purpose:**

To explore the epidemiology of *C. difficile* colonization in LTCFs, identify predisposing factors and describe its impact on healthcare management.

**Data Sources:**

PubMed, Embase and Web of Science (up to June 2014) without language restriction, complemented by reference lists of eligible studies.

**Study Selection:**

All studies providing extractable data on the prevalence of toxigenic *C. difficile* colonization among asymptomatic residents in LTCFs.

**Data Extraction:**

Two authors extracted data independently.

**Statistical Methods:**

The pooled colonization estimates were calculated using the double arcsine methodology and reported along with their 95% random-effects confidence intervals (CIs), using DerSimonian-Laird weights. We assessed the impact of patient-level covariates on the risk of colonization and effects were reported as odds ratios (OR, 95% CI). We used the colonization estimates to simulate the effective reproduction number R through a Monte Carlo technique.

**Results:**

Based on data from 9 eligible studies that met the specified criteria and included 1,371 subjects, we found that 14.8% (95%CI 7.6%-24.0%) of LTCF residents are asymptomatic carriers of toxigenic *C. difficile*. Colonization estimates were significantly higher in facilities with prior CDI outbreak (30.1% vs. 6.5%, p = 0.01). Patient history of CDI (OR 6.07; 95% CI 2.06–17.88; effect derived from 3 studies), prior hospitalization (OR 2.11; 95% CI 1.08–4.13; derived from 3 studies) and antimicrobial use within previous 3 months (OR 3.68; 95% CI 2.04–6.62; derived from 4 studies) were associated with colonization. The predicted colonization rate at admission was 8.9%.

**Conclusion:**

Asymptomatic carriage of toxigenic *C. difficile* represents a significant burden in LTCFs and is associated with prior CDI outbreaks in the facility, a history of CDI, prior hospitalization and antimicrobial use. These findings can impact infection control measures at LTCFs.

## Introduction

*Clostridium difficile* is the leading cause of nosocomial diarrhea and particularly affects the elderly population [1]. The Centers for Disease Control and Prevention characterizes *C. difficile* as a pathogen of significant public health interest which warrants urgent attention [2]. This is not surprising, given that *C. difficile* infection (CDI) accounted for 12.1% of healthcare associated infections in the U.S. [3], leading to historic highs in related deaths and healthcare costs [4,5]. Even though there is supporting evidence that asymptomatic carriers contribute to disease transmission of epidemic and non-epidemic strains in long-term care facilities (LTCFs), screening of asymptomatic carriers is not mandated by current infection control guidelines [6,7]. The ratio of asymptomatic colonized patients to patients with *C. difficile* diarrhea is estimated to be ∼4:1 in hospital facilities [8,9]. Furthermore, there is supporting evidence that colonized individuals contribute to disease spread [10]. In a seminal report, 84% of patients with nosocomial acquisition of a *C. difficile* strain had the same strain introduced earlier by an asymptomatic ward admission [10]. Approximately one out of five colonized patients would subsequently develop *C. difficile* diarrhea [11].

Notably, data regarding CDI in the elderly in LTCFs [12–14] is sparse. Even less data exists regarding *C.difficile* colonization. As a result, the epidemiology of toxigenic *C. difficile* in this population is largely unknown, including the factors associated with colonization status and the subsequent risk of infection. In this context, we systematically reviewed the published evidence to quantify pertinent data and provide a frame for studying the epidemic of *C.difficile* in LTCF residents.

## Methods

We searched PubMed, Embase and Web of Science (up to June 2014) without language restriction for pertinent articles on colonization, using the single term “difficile”. This meta-analysis was conducted according to the PRISMA guidelines [15] (S1 PRISMA Checklist). After title and abstract screening, two of the contributing authors (IMZ and EEP) independently determined article relevance and those deemed eligible were retrieved in full-text to be included in the analysis. Additional studies were sought through the reference lists of eligible studies. Unpublished data, editorial material and studies published solely in abstract form were not considered. The sole eligibility criterion was to report extractable data on toxigenic *C. difficile* colonization (primary outcome) in LTCFs.

Colonization (endemic prevalence) was calculated by dividing the number of residents who tested positive (rectal swabs or stool samples) for toxigenic *C. difficile*, by the total number of asymptomatic residents screened in the study. Patients who were symptomatic at screening were excluded from the at-risk population. Additional data retrieved and reported from eligible studies included publication year, setting, location, resident demographics and method of isolation. All information was extracted independently by two reviewers (PDZ, EEP). The two reviewers used the same data extraction form. After comparing the extracted data, a consensus was reached in cases of discrepancy.

We used the Newcastle-Ottawa Quality Assessment Scale (NOS) for observational data to rate the methodological quality of the eligible studies [16,17]. In brief, the NOS tool rates selection, comparability and outcome using a quality score ranking [18]. An individual study can have a maximum of 8 quality points. Studies that were rated with 4 or more quality points were deemed to be adequate in quality, and had their relevant data extracted and pooled. We defined toxigenic *C. difficile* colonization as toxin isolation and/or growth of a toxigenic strain of *C. difficile* from stool samples or rectal swabs in asymptomatic residents[19].

The combined effects were summarized using random-effects confidence intervals [20], after stabilizing variances via the double arcsine transformation to avoid assigning undue large weights for studies with extremely low (close to zero) or extremely high (close to 100%) prevalence [21]. We measured statistical heterogeneity using the tau-squared statistic (τ^2^) [22] and assessed publication bias with the Egger’s regression test [23]. A meta-regression technique was used to further explain heterogeneity. We explored the association of previous CDI, prior hospitalization, antibiotic use or proton pump inhibitor (PPI) use with *C. difficile* colonization. When heterogeneity was present, random effects models (RE) may be more appropriate and yield conservative estimates. However, in the absence of significant heterogeneity, a fixed-effects (FE) model was preferred to pool the relative effects. The pooled (unadjusted) effects were calculated and presented as Odds Ratios (95% Confidence Intervals), with an OR>1 denoting an increased risk of colonization.

We applied the Monte Carlo technique to predict the effective reproduction number R and colonization with *C. difficile* at LTCF admission (admission prevalence). In order to do this, we utilized our pooled colonization estimates on the model by Austin *et al*. [24] to simulate transmission dynamics [25], assuming that *C. difficile* residents remain colonized throughout their stay. The effective reproduction number R is derived by solving the following equation: R = [y_p_ (1+δφ)-(δ+1)φ]/[y_p_(1-y_p_)] [24], where R is equal to or greater than zero, δ is the proportional increase in length of stay for colonized patients, y_p_ is the endemic prevalence and φ is the admission prevalence. The effective reproduction number is the key parameter in infectious disease dynamics, and reflects both the transmission potential of disease and the performance of infection control measures. The effective reproduction number provides the average number of secondary cases arising per each infected individual. For example, if R = 2, then this means a single *C. difficile* carrier would produce, on average, two secondary cases. If R<1, then this means transmission alone is unable to sustain the endemic.

We used the Stata v13 software package (Stata Corporation, College Station, TX, USA) and MATLAB (Mathworks, Natick, MA,USA) to perform the data analysis and construct the pertinent graphs.

## Results

**Fig 1:**
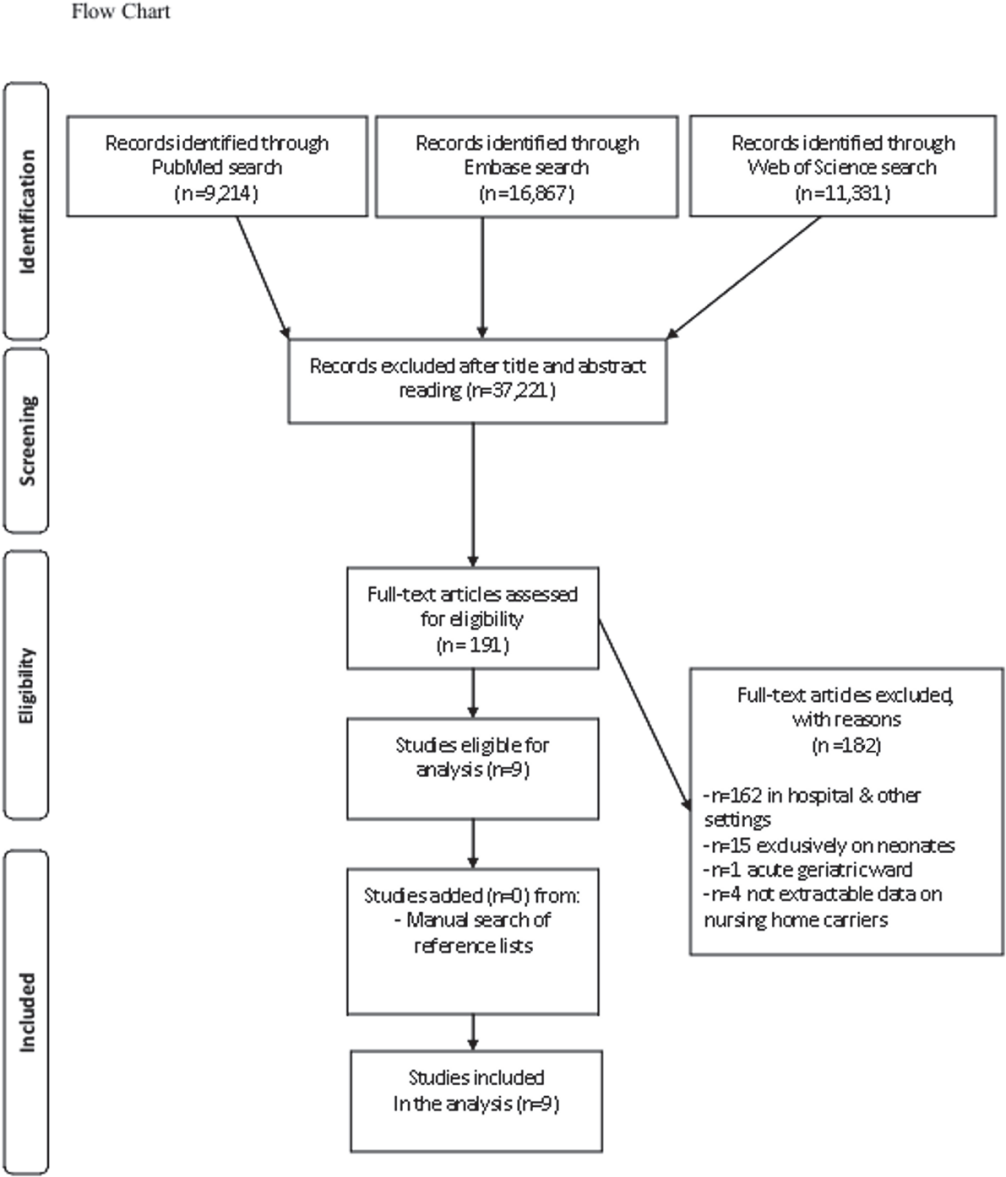
Flow chart.

The databases search (last accessed on June 20, 2014) yielded 9,214 articles from PubMed (1925–2014), 16,867 articles from EMBASE (1947–2014) and 11,331 from Web of Science (1925–2014). From 191 studies reviewed in full text, 9 were considered to meet the inclusion criteria for the final analysis. Among the remaining studies, 182 were excluded because they referred to settings other than LTCFs (178 studies) or did not provide extractable data on *C. difficile* colonization (4 studies) [26–29]. A complementary search of reference lists did not yield any additional studies that would be eligible for analysis (Fig. 1-Flow chart).

The 9 eligible studies [7,30–37] were published between 1990–2012 and originated from the USA (6 studies), Canada (1 study) and Europe (2 studies). The mean age reported across studies varied from 70 to 85 years (median 88.5), with a female predominance (median 69%, range 0–81%). All studies implemented a multistep process to detect toxigenic *C. difficile* that included anaerobic culture, cell culture cytotoxicity assays (CCCA) and enzyme immunoassay (EIA) for *C. difficile* toxins A and B, and PCR. The detailed characteristics of the included studies are seen on Table 1.

**Table 1:** Summary data of individual studies.

Study, year	Country	Setting	Preceding outbreak of CDI	Method of *C.dificile* detection	N	Mean Age (years)	Female gender(%)	n (%) colonized
**All studies**	**6/9 (67%) USA**	**21 LTCFs**	**4/9 (44%)**	**All used multi-step detection**	**1371**	**70 to 85**	**Range 0–81%**	**17014.8 (95% CI 7.6–24.0)†**
**Arvand, 2012[30]**	Germany	11 LTCFs	No	Culture and toxigenicity confirmation with EIA and PCR	239	85 (38–100)	78%	9 (3.8%)
**Ryan, 2010 [31]**	Ireland	1 LTCF (2 wards)	No	Culture and PCR for detection of *C.difficile* and toxigenicity confirmation with EIA for toxin A/B and PCR	100	83	69%	7 (7.0%)
**Riggs, 2007 [7]**	USA, OH	1 LTCFs (2 wards)	Yes	Culture and toxigenicity confirmation with EIA for toxins A/ B	68	70±12	0%	35 (51.5%)
**Rivera, 2003 [32]**	USA, OH	1 LTCF (2 wards)	No	Culture and toxigenicity confirmation with EIA for toxins	42	NR	NR	2 (4.8%)
**Simor, 1993 [34]**	Canada	2 LTCF (9 wards)	No	Culture and toxigenicity confirmation with CCCA	489	NR	NR	57 (11.7%)
**Walker, 1993[33]**	USA, MN	2 LTCFs	No	Culture and toxigenicity confirmation with CCCA	225	84.2±9.9	81%	9 (4.0%)
**Bennett**,**1992 [35]**	USA, MD	1 LTCF	Yes	Culture and toxigenicity confirmation with CCCA	59	75(28–99)	66%	23 (38.9%)
**Larson, 1991 [36]**	USA, MD	1 LTCF (5 wards)	Yes	Culture and toxigenicity confirmation with CCCA	100	NR	NR	23 (23%)
**Kerr, 1990 [37]**	USA, MT	1 LTCF	Yes	Culture and toxigenicity confirmation with CCCA	49	85(38–100)	71%	5 (10.2%)

CDI = *C.difficile* infection, CCCA = cell culture cytotoxicity assays, EIA = enzyme immunoassay, LTCF = long-term care facility, NR = not reported, †pooled random-effects estimate

### Facility-level data

All studies were deemed to be of adequate quality in order to be included in the analysis (S1 Appendix—Quality assessment). Across the 9 included studies that enrolled a total of 1,371 residents, the pooled colonization with toxigenic *C. difficile* was 14.8% (95%CI 7.6–24.0). Heterogeneity was high (τ^2^ = 0.11) and there was no evidence of small study effects across studies (Egger’s bias 4.17, p = 0.26). Four studies reported a preceding outbreak (Table 1). A “Preceding CDI outbreak” was a study reported outcome to describe an increased number and clustering of CDI cases within the specific facility setting that preceded the conduction of the study. Although the definition is not standardized, the reported outbreaks comply with the general term adopted by the World Health Organization that “a disease outbreak is the occurrence of cases of disease in excess of what would normally be expected in a defined community, geographical area or season” [38]. Across facilities with preceding CDI outbreaks (4 studies, 276 residents), the estimated asymptomatic carriage was higher (30.1%; 95%CI 14.7–48.2) compared to those without (6.5%; 95%CI 3.3–10.6 across 5 studies and 1095 residents, p = 0.01). In a meta-regression analysis, model heterogeneity was reduced from τ^2^ = 0.17 to τ^2^ = 0.06 after accounting for previous facility-level outbreak.

### Patient-level data

**Fig 2:**
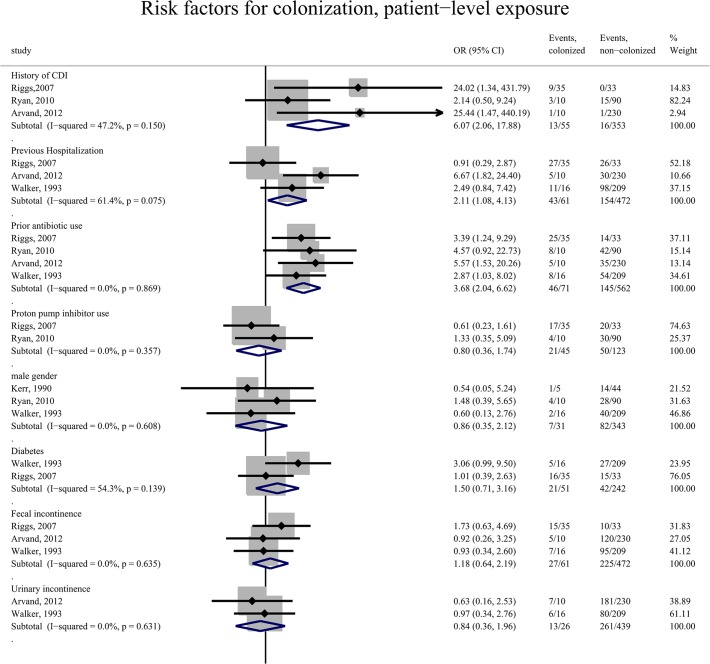
Forest plot of resident characteristics associated with *C. difficile* colonization.

Colonization with *C. difficile* was associated with history of CDI (OR 6.07; 95% CI 2.06–17.88; derived from 3 studies totaling 408 residents), antibiotic use within previous 3 months (OR 3.68; 95% CI 2.04–6.62; derived from 4 studies totaling 633 residents), and prior hospitalization within the past 3 months to 1 year (OR 2.11; 95% CI 1.08–4.13; derived from 3 studies totaling 533 residents) (Fig. 2). Colonized individuals did not differ significantly from those that were non-colonized in terms of age (pooled mean difference -0.6 years, p = 0.5), gender and comorbidities (including diabetes and urinary/fecal incontinence) across studies that compared the two groups (Fig. 2). There was also no difference between groups with respect to median length of stay in LTCF (526.5 *vs*.573.5 days, p = 0.19 in [7] and 55 *vs*. 44 days in [31], p = 0.54) and overall mortality (single study reported pertinent data, 23% *vs*. 15%, p = 0.42) [7].

### Simulation data

We estimated the effective reproduction number and admission colonization rate at LTCFs by taking into account our pooled colonization estimates (for parameter y_p_) and the percentage increase in length of stay for colonized over non-colonized individuals (parameter δ). Two studies [7,31] that provided pertinent data regarding length of stay, indicated that there is no significant difference between colonized and non-colonized residents, and consequently the parameter δ would be close to 0.

**Fig 3:**
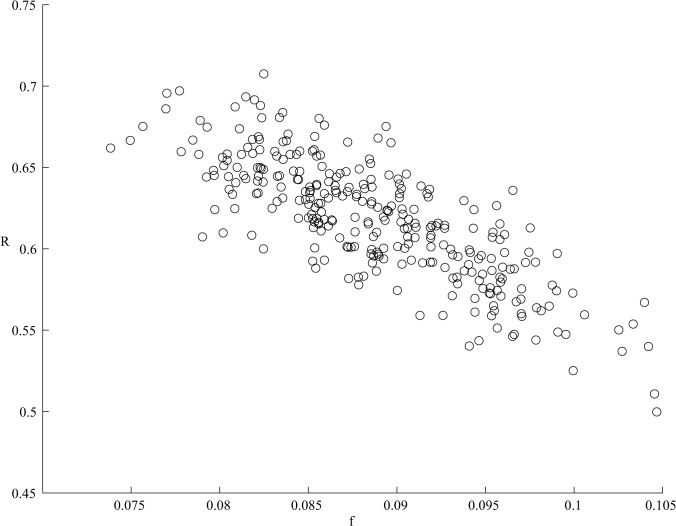
Simulated effective reproduction number (R) and admission prevalence (φ) pairs in replication sample (300 replications).

We simulated the effective reproduction number R using our upper and lower 99% precision estimates (5.9% to 27%) as the limits for y_p_, and δ in a range of 25% [7] to -8% [31]. We assumed a uniform distribution (the most conservative approximation over a normal distribution) for parameters y_p_ and δ. Colonization at admission in LTCFs is unknown, and was simulated at a range of 0–100% from uniform distribution. Transmission dynamics allow only for positive (or zero) reproduction numbers and only model replicates that satisfy the condition that R≥0 apply. We have drawn a random 100-observation sample and simulated the model 300 times. The mean for predicted colonization at admission (φ) was 8.9% (Fig. 3 displays the predicted R, φ pairs in replication data). The predicted mean reproduction number R was 0.62 (95% prediction interval 0.55–0.69). In other words, these estimates indicate that on average, two new *C. difficile* carriers will arise in long-term care facilities for every three individuals colonized at admission. As R is below unity, *C. difficile* colonization will be sustained within a long-term care facility mainly due to admission of carriers and, to a lesser extent, due to secondary cases.

## Discussion

This systematic assessment focuses on the epidemiology of *C. difficile* in LTCF. First, we estimated that over a tenth of asymptomatic LTCF residents harbor toxigenic *C. difficile* during their stay. Also, our results correlated antibiotic use over the previous 3 months, and previous CDI with increased risk for colonization in this population. Moreover, in the setting of a previous outbreak, nursing facilities have a significantly higher burden of colonized residents. Finally, we used these estimates to perform a simulation analysis that yielded a low reproduction number, a finding that underscores the significance of colonization with toxigenic strains at admission. The findings of simulation analysis indicated that on average two new *C. difficile* carriers will arise in LTCFs for every three individuals colonized at admission.

Colonization with toxigenic *C.difficile* in LTCFs (14.8%) may be considered comparable to the acute-care setting. Colonization varied between 8–20% in hospital cohort studies [39–41]. The risk factors associated with colonization are similar to those described for CDI in the elderly. For example, a population cohort study that enrolled 79,039 subjects aged ≥65 years found that antibiotic exposure and prior hospital admission were independently associated with CDI, whereas PPIs had no significant effect. Care home residents had twice the associated risk for CDI compared to those living in their own home [42]. Furthermore, there is supporting evidence that *C. difficile* epidemiology shifts from being a hospital-based infection to a LTCF-based infection, as nearly half of cases diagnosed in hospital had acquired *C.difficile* in LTCFs [43].

The clinical spectrum of *C. difficile* ranges from asymptomatic disease to fulminant colitis. It is postulated that asymptomatic disease is associated with a strong immune response to *C. difficile* toxins whereas patients with severe illness and impaired immune response develop *C. difficile* diarrhea [44]. However, asymptomatic carriers are a source of infection that remains undetected and likely contributes to disease spread unless surveillance and infection control methods are implemented. Notably, there is no evidence of immune protection against *C. difficile* colonization [44], rendering the whole LTCF population vulnerable in endemic settings. The elderly have increased susceptibility to acquire *C. difficile* due to immune senescence, changes in fecal flora, impaired antibody response to *C. difficile* toxins as well as the presence of comorbid conditions, use of antibiotics and prior hospitalizations [45].

*C. difficile* is a transmissible disease in LTCF and transmission is likely to occur by direct spread from the hands of personnel, fomites and LTCF environment and is facilitated by the fact that residents share quarters and facilities (for sleeping, eating and toileting) [46–48]. For example, the importance of the inmate environment in the spread of CDI is highlighted by a previously reported outbreak of *C. difficile* in a LTCF that was related to the re-use of contaminated electronic thermometers [46]. However, ward-based contact between symptomatic subjects accounts only for the minority of CDI cases [49] and asymptomatic carriers are now considered a potential source for CDI and reservoir for *C. difficile* [50] that might contribute to transmission [51–53]. Whole genome sequencing of *C. difficile* strains indicated that transmission attributed to symptomatic patients accounts only for a minority of CDI acquisitions and that asymptomatic carriers contribute to the chain of transmission [52]. Molecular genotyping has showed that one out of four CDI isolates are related to isolates from previously documented asymptomatic carriers and supports the hypothesis that infection control policies targeting carriers may prevent CDI cases [53]. The potential role of asymptomatic carriers is further supported by the finding that a prolonged carrier state and shedding are seen for patients that have been treated for CDI [54] and the report that *C. difficile* strains are recovered on skin and environmental samples, most of which were identical to the isolates of stool samples from carriers [7].

More than 1.4 million reside in LTCFs in U.S., a figure that corresponds to 2.8% of the population over 65 and 10.2% of the population over 85 [55]. Consequently, based on our estimations, ∼200,000 LTCF residents may be asymptomatic carriers of *C. difficile* toxigenic strains, where the role of the asymptomatic carrier is not evaluated in detail and surveillance for *C. difficile* colonization is not recommended [6]. This contrasts to the management of pathogens such as methicillin-resistant *Staphylococcus aureus* (MRSA) or vancomycin-resistant enterococci (VRE) [17,25,56,57], as carriers of MRSA and VRE are targeted through various infection control programs that include surveillance, isolation and decolonization [58]. Further studies are needed to determine the feasibility and effectiveness of similar infection control policies for *C. difficile* colonized patients in LTCFs. Importantly, our analysis indicates that lack of infection control policies should be re-evaluated and signify that LTCFs are a source for toxigenic *C. difficile* that may sustain the epidemic.

It should be noted that the limited potential for isolating infected residents, combined with the difficulty in removing spores from the environmental surfaces with the commonly used detergent-based cleaners [59] and the ineffectiveness of alcohol-based waterless hand sanitizers to remove *C. difficile* [60], render infection control of *C. difficile* challenging [45,61]. Nevertheless, as use of antimicrobial agents was associated with *C. difficile* colonization, antibiotic stewardship programs [62] deserve consideration as parts of *C. difficile* prevention policies. Also, the introduction of rapid and sensitive screening to avoid the long turn-around time of culture methods [63] may facilitate early identification and isolation of asymptomatic carriers soon after LTCF admission and may contribute to the control of the epidemic. For example, a recent transmission model in the hospital setting showed that screening and isolation of asymptomatic carriers would reduce both the number of new colonization cases up to 50% and CDI cases by 25% for various scenarios tested at the ward level [64]. Specific research targeting LTCF residents is needed to evaluate feasibility, outcomes and cost.

Importantly, our simulation data showed that LTCF transmission alone may be unable to sustain *C. difficile* colonization, given the predicted low (<1) reproduction numbers. Consequently, the admission of colonized subjects will play the important role in sustaining the epidemic and support the position that efforts to constrain *C. difficile* transmission should not be limited to LTCFs. A previous epidemiological model in hospitals yielded a low reproduction number (median R = 1.04, range 0.55–1.99) and concluded that admission of carriers plays an important role, whereas transmission within the ward alone cannot sustain *C.difficile* epidemics [65]. These estimations are in line with our findings and underscore a comparable transmission pattern across various healthcare settings, including hospitals and LTCFs. More than 50% of LTCF admissions derive from acute care hospitals and hospital-based facilities [66] which are considered the main source for toxigenic *C. difficile* [1]. 24.8% of nursing residents require at least one hospitalization during their stay [67], and a significant number of asymptomatic carriers will be introduced in the nosocomial setting. These figures mandate a combined effort to control the epidemic in both acute care hospital and LTCFs.

Our analysis is limited by the relative paucity and quality of the individual studies on this topic. Time trends, different local epidemiology (including the impact of virulent strains), demographic differences and confounding variables cannot be accounted for. Publication bias was not detected by Egger’s test, but caution is warranted as less than ten studies were available for analysis. Moreover, transmission dynamics were modeled using the available data and the most conservative assumptions, in lieu of actual observations for admission prevalence that lack in LTCF settings. Also, the use of PPIs was not associated with *C. difficile* colonization. However, due to limited data and unadjusted effects, caution is warranted when interpreting these associations, specifically for PPIs and prior contact with the healthcare system, factors known to increase the risk of CDI [68–70]. Finally, the effect of antimicrobial agents is not adjusted for different classes and confounding where previous CDI may be present. A multivariable analysis to adjust for influential confounders would be ideal, but is precluded due to the absence of individual patient data (as IPD meta-analysis) or data stratified by parameters of interest.

The contribution of colonized patients to the burden of symptomatic CDI and the impact of infection control policies has not been clearly established. The effectiveness and cost of interventions to reduce *C.difficile* in long-term care facilities will be derived solely from prospective trials. Surveillance of asymptomatic carriers would increase health care expenditure, adding the cost of screening and contact precautions. However, if it reduces the risk of CDI, these precautions may prove cost-effective, given that each CDI case adds an average excessive cost of ∼$27,000 in health care [71].

In summary, this analysis described the burden of asymptomatic *C. difficile* colonization in LTCF facilities and the potential implications in *C. difficile* epidemiology. The burden of colonization increases for settings with prior CDI outbreak, and colonization at admission is expected to be a major determinant of disease spread. The current practice underestimates the importance of the *C. difficile* colonization in LTCFs and in view of these findings, control measures may not be limited to symptomatic patients but may extend to colonized residents harboring toxigenic strains. Such policy will require implementation of screening and surveillance programs similar to other drug resistant pathogens to constrain pathogen transmission.

## Supporting Information

S1 PRISMA Checklist PRISMA checklist. (DOC)

S1 Appendix Quality assessment. (DOC)
