# Supplementary material for: Asymptomatic Carriers of Toxigenic C. difficile in Long-Term Care Facilities: A Meta-Analysis of Prevalence and Risk Factors
Source: PLoS One. 2015 Feb 23;10(2):e0117195. doi: 10.1371/journal.pone.0117195 (PMC4338134; doi:10.1371/journal.pone.0117195)
Supplement: S1 Appendix — (DOC) [file pone.0117195.s002.doc]

| Study | Selection | | | | Comparability | Outcome | | | Score |
| --- | --- | --- | --- | --- | --- | --- | --- | --- | --- |
|  | Representativeness of the exposed cohort | Selection of the non-exposed cohort | Ascertainment of exposure | Demonstration that outcome of interest was not present at start of study | Comparability of cohorts on the basis of the design or analysis | Assessment of outcome | Follow-up long enough for outcomes to occur | Adequacy of follow-up of cohorts |  |
| Arvand | NR | NA | ★ | NA | NA | ★ | ★ | ★ | 4 |
| Ryan | - (57.1%) | NA | ★ | NA | NA | ★ | ★ | ★ | 4 |
| Riggs | (100%) | NA | ★ | NA | NA | ★ | ★ | ★ | 5 |
| Rivera | (100.0%) | NA | ★ | NA | NA | ★ | ★ | ★ | 5 |
| Simor | NR | NA | ★ | NA | NA | ★ | ★ | ★ | 4 |
| Walker | NR | NA | ★ | NA | NA | ★ | ★ | ★ | 4 |
| Bennett | NR | NA | ★ | NA | NA | ★ | ★ | ★ | 4 |
| Larson | (100%) | NA | ★ | NA | NA | ★ | ★ | ★ | 5 |
| Kerr | (100%) | NA | ★ | NA | NA | ★ | ★ | ★ | 5 |

NA=not applicable
